# Controlling Energy Flow in Perovskite Heterostructures Through Dimensionality and Phase Engineering

**DOI:** 10.1002/advs.202505971

**Published:** 2025-06-20

**Authors:** Danila A. Tatarinov, Alexander Schleusener, Roman Krahne

**Affiliations:** ^1^ Optoelectronics Group Italian Institute of Technology Genova Italy; ^2^ Departement of Chemistry and Industrial Chemistry Università Degli Studi di Genova Genova Italy

**Keywords:** dimensionality engineering, energy flow control, metal halide perovskites, optoelectronic properties, perovskite heterostructures, phase engineering, quantum confinement, structural dynamics

## Abstract

Metal halide perovskites have emerged as a transformative class of semiconductors, driving advancements in optoelectronics, photovoltaics, and sensing technologies. One of the key challenges in optimizing these materials for next‐generation devices is controlling the flow of energy within them, which is highly sensitive to structural and dimensional factors. Recent advances in phase and dimensionality engineering have opened new avenues for tailoring energy transport and excitonic behaviors in perovskite heterostructures. By controlling the dimensionality and tuning the phases of perovskites, it is possible to achieve enhanced efficiency, stability, and selectivity in energy transfer processes. This perspective explores the fundamental principles of energy flow in perovskites and related materials, highlighting how phase transitions and dimensionality control can be leveraged to design optimized heterostructures for cutting‐edge optoelectronic applications.

## Graphical abstract

Advances in phase and dimensionality engineering offer powerful strategies to modulate energy flow in perovskite heterostructures. This Perspective highlights how structural control at the nanoscale enables precise tuning of energy transport processes for next‐generation optoelectronic applications.

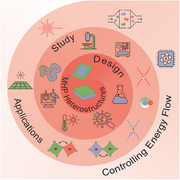

## 1 Introduction

Metal halide perovskites (MHPs) have rapidly emerged as one of the most promising classes of materials for next‐generation optoelectronic devices, offering exceptional optical properties, defect tolerance, and solution processability.^[^ ^1^, ^2^, ^3^, ^4^ ^]^ Their success in a wide range of applications, including high‐efficiency solar cells, light‐emitting diodes (LEDs), photodetectors, and lasers, has been driven by their unique electronic and excitonic properties.^[^ ^5^, ^6^, ^7^, ^8^ ^]^ However, a fundamental challenge that persists in perovskite‐based technologies is the precise control of energy flow, which governs key processes such as charge transport, exciton migration, and radiative recombination.^[^ ^9^ ^]^ The ability to control these processes is crucial for optimizing device performance, minimizing energy losses, and extending operational stability.^[^ ^10^, ^11^ ^]^

The efficiency of energy transport in perovskite materials is highly dependent on their structural and dimensional characteristics. Bulk 3D MHPs exhibit high charge carrier mobility and strong light absorption, making them ideal candidates for photovoltaic and light‐emitting applications.^[^ ^12^, ^13^ ^]^ However, they often suffer from instability due to phase transitions and environmental degradation. In contrast, lower‐dimensional perovskites offer enhanced stability but often exhibit limited charge transport due to quantum confinement effects.^[^ ^14^, ^15^, ^16^ ^]^ The interplay between these different dimensional regimes creates a vast design space for engineering energy transfer pathways, enabling new functionalities and enhanced performance in perovskite‐based heterostructures.

A particularly powerful approach to optimizing energy flow in perovskites is phase and dimensionality engineering, which allows for the strategic arrangement of different perovskite phases within a heterostructure to direct charge and exciton dynamics.^[^ ^17^, ^18^ ^]^ By incorporating dimensional confinement and leveraging phase interactions, it is possible to achieve a tailored energy flow that facilitates efficient charge separation or radiative recombination, depending on the specific application.^[^ ^19^, ^20^ ^]^ In this Perspective, we refer to the term “energy flow” as energy transfer, exciton diffusion, and charge carrier transport, as all these processes can be central to controlling the optoelectronic functionality of perovskite heterostructures. For instance, integrating quantum‐confined nanocrystals (NCs) with 2D layered metal halide perovskites (2DLPs) can enable energy‐cascading mechanisms that enhance light emission in LEDs, while embedding 2D perovskites as interfacial layers in 3D perovskite solar cells can improve carrier extraction and stability.^[^ ^21^, ^22^, ^23^, ^24^ ^]^ By exploiting the dependence of the band gap on the halide composition, lateral and vertical band gap engineering can be achieved that allows for funneling charge carriers to specific regions in a thin film or single microcrystal.^[^ ^25^, ^26^ ^]^ Furthermore, dynamic phase transitions in perovskites provide a unique opportunity for controlling energy transport in real time, opening pathways for switchable and tunable optoelectronic devices.

Despite these advances, several fundamental questions remain regarding the mechanisms that govern energy transfer in mixed‐dimensional perovskite systems.^[^ ^27^, ^28^ ^]^ Understanding how factors such as defect states, interfacial strain, and exciton binding energies influence charge carrier dynamics is essential for designing next‐generation materials with optimized energy flow properties.^[^ ^28^, ^29^ ^]^ Additionally, theoretical modeling and advanced spectroscopic techniques are crucial for elucidating the role of phase coherence, and charge separation processes in perovskite heterostructures.^[^ ^30^, ^31^, ^32^ ^]^

In this Perspective, we explore emerging strategies for controlling energy flow in low dimensional perovskite materials, with a focus on applications that require precise management of charge carrier dynamics. We put our emphasis on exciton dynamics, spatially variant band alignment, and quantum confinement effects, which are of great current interest for photocatalysis, energy harvesting, and photonic devices such as coherent light sources and single‐photon emitters. We begin by outlining fabrication strategies for perovskite heterostructures via phase and dimensionality engineering, discuss how these methods lead to novel material properties and enable efficient energy modulation, and attempt charting a roadmap for next‐generation optoelectronic devices. In this view, we include other emerging materials that can be combined with perovskites, or which offer similar optoelectronic and structural versatility.

1 **Box 1: Dimensionality Control and Structural Variety in Metal Halide Perovskites**

MHPs can form 3D or lower‐dimensional structures depending on the type of constituent ions and spacer groups.^[^ ^33^ ^]^ 3D MHPs are characterized by the general formula ABX_3_, where the metal cation “B” is coordinated to six nearest‐neighbor anions “X,” while the organic (inorganic) cation “A” occupies a position within a cavity formed by eight corner‐sharing BX_6_ octahedra that form a continuous perovskite lattice.^[^ ^34^ ^]^ Dimensionality reduction of perovskite crystals can unlock new properties and applications by inducing dielectric or quantum confinement.

**Figure 1:**
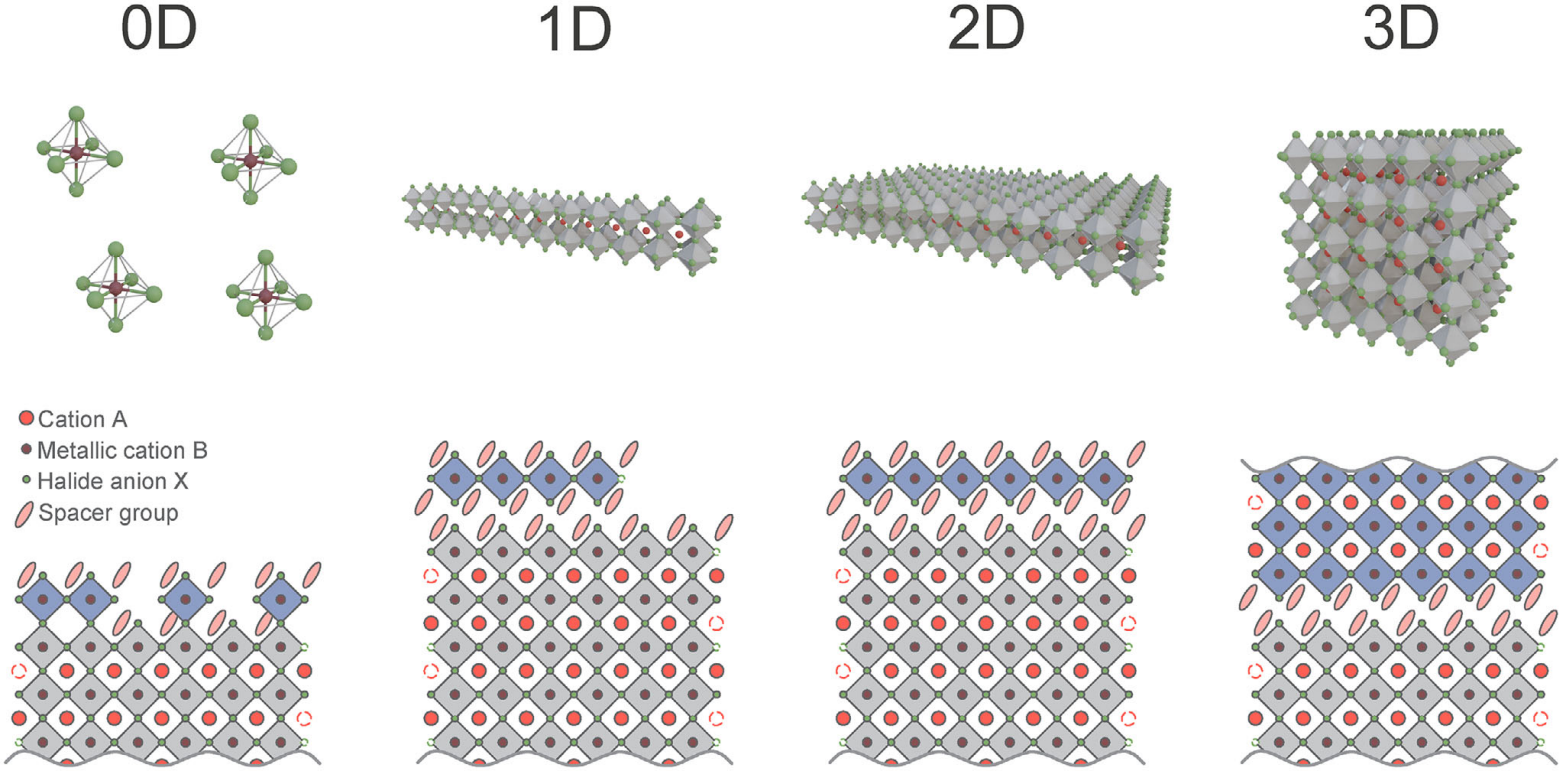
Typical crystal structures of metal halide perovskites (upper panel) and possible configurations of heterostructures with mixed dimensionality (bottom panel), from left to right: 0D; 1D; 2D; and 3D.

Low‐dimensional MHPs, which can adopt various stoichiometries, are classified into 2D, 1D, or 0D types.^[^ ^35^ ^]^ 2D organic‐inorganic hybrid structures originate from 3D perovskites by removing inorganic layers along specific crystallographic directions, such as (100), (110), or (111) (**Figure** **1**). The optical properties, including emission wavelengths and exciton binding energies, can be fine‐tuned by varying the number of octahedral layers between organic spacers, where the number of layers (n) can range from 1 to infinity. These structures are commonly represented by the formula A“_m_A_n‐1_B_n_X_3n+1_, where m depends on the perovskite structure type, A” is the spacer cation (often a bulky organic ligand), A is the cavity cation, and n indicates the number of octahedral layers between the organic spacers.^[^ ^36^ ^]^ In comparison to 3D perovskites, their 2D systems generally exhibit higher exciton binding energies and excitonic behavior, making them highly suitable for applications in photonics.

1D perovskite structures, in contrast to 2D, are characterized by shorter charge diffusion lengths and higher aspect ratios. These structures can also exhibit a variety of stoichiometries and are typically composed of chains formed by PbX₆ octahedra that are corner‐, edge‐, or face‐sharing, separated by organic cations. The structural configuration of these chains depends on the nature of the A‐site cation, with certain cations enhancing water/humidity resistance due to the passivation of the octahedra provided by the organic spacers. Further reducing dimensionality, 0D structures, such as A₄PbX₆, consist of isolated PbX₆ octahedra or lead halide clusters, often stabilized by large organic cations.^[^ ^37^ ^]^ Unlike their higher‐dimensional counterparts, 0D perovskites demonstrate suppressed ion migration and reduced hysteresis in photovoltaic applications, leading to more stable optoelectronic devices.

Dimensionality control in MHPs plays a crucial role in tuning their optoelectronic properties and expanding their application range. By precisely engineering the dimensionality of the perovskite structures, it is possible to optimize their behavior for specific devices such as solar cells, LEDs, and photodetectors, for example by designing the electrical confinement landscape or increasing the robustness of their surfaces.^[^ ^38^, ^39^ ^]^ Toward the design of charge carrier confinement, in 2D perovskites, the number of octahedral layers between the organic spacers directly impacts the band gap, exciton binding energy, and emission wavelength, which can be tailored for optimal device performance. Similarly, in 1D perovskites, controlling the length and connectivity of PbX₆ octahedral chains influences charge diffusion lengths and charge transport efficiency, making them suitable for applications requiring high aspect ratios and fast charge movement. In 0D perovskites, the complete separation of PbX₆ octahedra by organic cations leads to highly localized electronic states, affecting the energy level structure, carrier relaxation, recombination dynamics, and charge transport properties.

In mixed‐dimensional systems, the different dimensionalities (0D, 1D, and 2D) are combined in a single system, and their interfaces constitute heterostructures. Such a mixed‐dimensional approach provides additional opportunities for tailoring energy transfer and charge carrier localization and broadens the design space for increased device functionality.^[^ ^40^, ^41^ ^]^ The interplay between dimensionality and phase, therefore, opens new avenues for optimizing the performance of perovskite‐based materials in diverse optoelectronic and photonic applications. We note that in this perspective we refer to dimensionality engineering in terms of reduced octahedral connectivity within the perovskite lattice, which is different from the physical size reduction of 3D MHPs in all three spatial dimensions that gives rise to nanocrystals or quantum dots. Quantum dots structurally retain a 3D octahedra connectivity, while 0D perovskites are materials featuring isolated metal‐halide octahedra with no extended structural connectivity, leading to fundamentally different electronic properties.

1 **Box 2: Architecture Control in Mixed‐Dimensional Metal Halide Perovskites Heterostructures**

**Figure 2:**
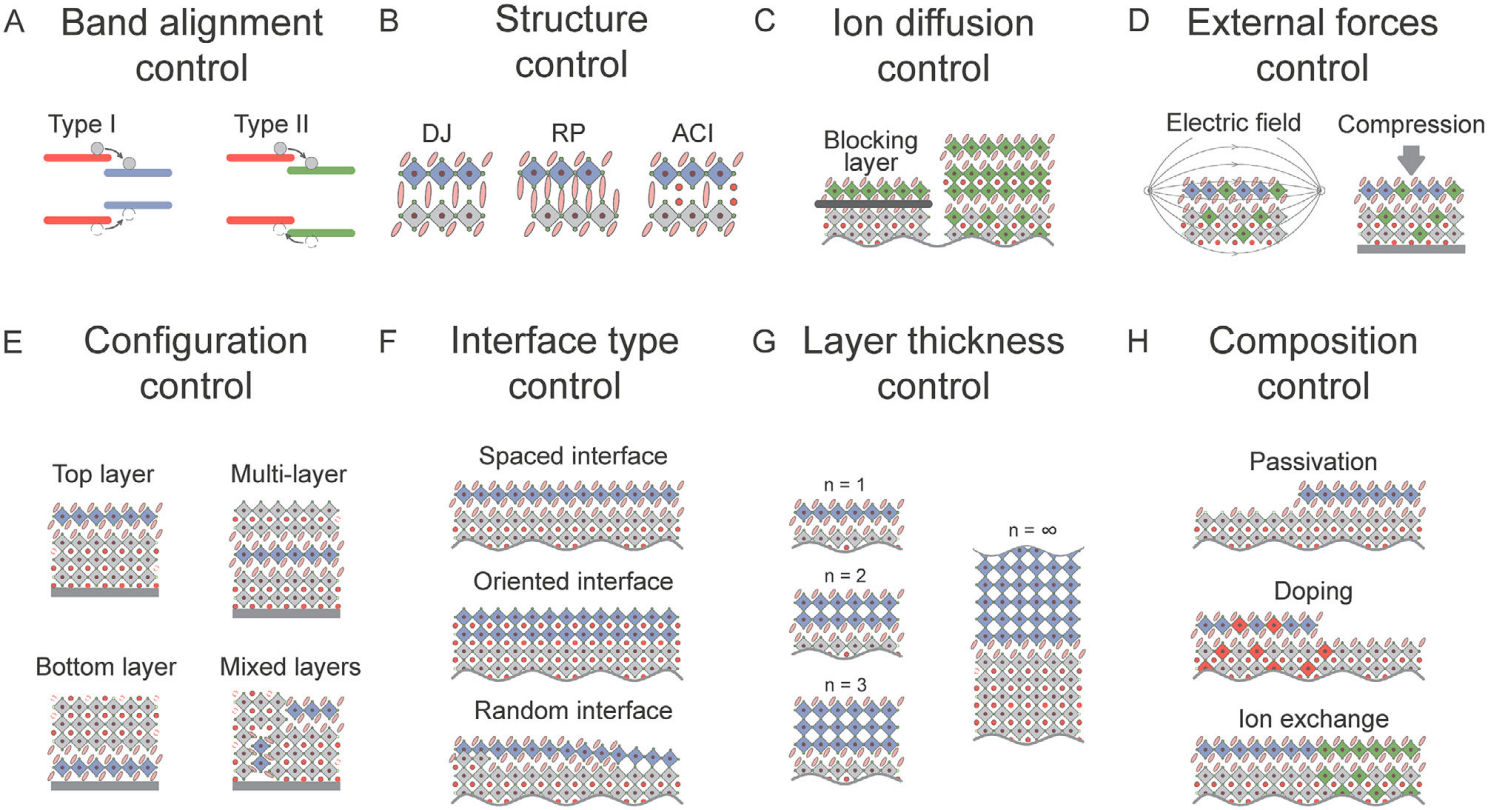
Energy flow control approaches in mixed‐dimensional metal halide perovskites. A) Type I and II interfacial band alignments that define the energy and charge transfer pathways, in which each configuration promotes distinct mechanisms of charge movement across the interface; B) Structure control involves modifying the perovskite phase type, such as the Dion–Jacobson (DJ) phase, Ruddlesden–Popper (RP) phase, and alternating cation (ACI) phase, to tailor the material's properties; C) Ion diffusion control, and blocking layers to prevent interfacial ion diffusion and structural homogenization; D) External force control, including various approaches that apply external fields or strain to tune the material properties; E) Configuration control, involving different schemes for the arrangement of perovskite layers; F) Interface type control, where the interaction between materials can be mediated by non‐bonding spacer groups with a narrow van der Waals gap, an epitaxial interface with matched lattice constants and coherent atomic bonding, or a random interface where lattice mismatch and atomic bonding lead to defects like voids, dangling bonds, or interfacial trap states; G) Layer thickness control, achieved by varying the number of octahedral layers between organic spacers; H) Composition control, involving interface passivation, structural doping with various ions, and ion exchange to tune both cation and anion perovskite compositions.

Along with dimensional engineering, various strategies for energy flow and charge transfer control in MHPs further enhance the potential of these materials for advanced optoelectronic and photonic applications.^[^ ^42^ ^]^ **Figure** **2** illustrates various control strategies concerning energy flow in mixed‐dimensional MHPs for optimizing their properties for specific applications.

Heterostructures in materials with different, type‐I or type‐II, interfacial band alignments can be employed to tailor charge and energy transfer processes at the material interface (Figure 2a). In type‐I alignment, the conduction and valence bands of one material are fully encompassed by the other, and thus charge carriers (including excitons) relax into the lower bandgap region, which can be exploited for funneling energy across the junction. In the type‐II alignment, the bands are staggered, fostering spatial separation of charge carriers across the heterojunction, which can be advantageous for photocatalysis and energy harvesting. In addition, structural engineering enables the tailoring of the material's phase to tune electronic properties, stability, and overall performance. In this respect, differences in halide or metal cation composition, as well as different organic phases, including alternating cation in the interlayer space (ACI) perovskites with the formula A′A′′_n_Pb_n_X_3n+1_ (n ≥ 1), quasi‐layered Dion‐Jacobson (DJ) perovskites with the formula A′′A_n–1_Pb_n_X3_n+1_ (n ≥ 1), and Ruddlesden–Popper (RP) perovskites with the formula A′_2_A_n–1_Pb_n_X3_n+1_ (n ≥ 1) can be used. Here A, A′, and A″ represent small organic or inorganic cations or large spacer monovalent and divalent cations, respectively (Figure 2b). This structural diversity provides a rich design space for perovskite‐based materials to tailor their optoelectronic properties.

Toward stable interfaces, ion diffusion control is crucial, and specific blocking layers have been introduced to prevent undesired ion migration into other phases, thereby stabilizing the perovskite structure and reducing degradation (Figure 2c).^[^ ^43^ ^]^ Ion migration at the heterointerface also plays a critical role, as it can induce dynamic changes in band alignment, cause interfacial degradation, or alter carrier dynamics over time. A thorough understanding and control of these ionic processes is essential, as they often precede and dominate the long‐term stability and functionality of the heterostructure, potentially overshadowing the intended electronic and optical effects.^[^ ^44^, ^45^, ^46^ ^]^ The application of external forces, such as electric fields or mechanical strain, provides another toolbox to tune material properties by altering the crystal structure, electronic states, or optical properties, offering a dynamic means of controllingthe behavior of the system (Figure 2d). For instance, in vertically stacked [(*n* = 3)/(*n* = 1)] quasi‐2D perovskite heterostructures prepared via mechanical exfoliation, time‐resolved photoluminescence, and transient absorption microscopy revealed efficient interfacial exciton dissociation and ultrafast hole transfer within tens of picoseconds, highlighting the sensitivity of interfacial charge dynamics to structural modulation.^[^ ^47^ ^]^ The arrangement of perovskite layers, whether in a vertical or horizontal orientation, can affect the material's charge transport and optical characteristics. This approach can be used both to optimize the electrical properties (for example for charge extraction) and to improve environmental stability (Figure 2e). The core concept involves forming 2D layers on the surface or at the bottom of 3D perovskite grains, which passivates defects and protects the surface from environmental degradation.^[^ ^48^ ^]^ Another method to fabricate 2D/3D heterostructures is through in situ cation exchange by depositing a ligand salt solution onto pre‐synthesized 3D perovskite thin films, converting the 3D surface into 2D perovskites.^[^ ^49^ ^]^

To control the heterostructure formation process, various strategies have been developed, including solvent, ligand, and annealing engineering. Solvent engineering, such as anti‐solvent dripping, allows precise control over crystal nucleation and 2D layer thickness.^[^ ^50^ ^]^ Ligand engineering focuses on modifying the organic spacer's structure, which affects the dimensionality and stability of the heterostructure.^[^ ^51^ ^]^ Annealing engineering involves thermal treatments to modulate phase distribution and improve crystallinity, while processing engineering, like layer‐by‐layer deposition, enables fine‐tuning of 2D layer thickness and uniformity.^[^ ^52^ ^]^

The nature of the interaction between materials at the interfaces also plays a critical role in the behavior of the heterostructure. Whether through non‐bonding spacer groups with narrow van der Waals gaps, epitaxial interfaces with matched lattice constants, or random interfaces leading to defects, each type influences charge recombination, exciton transport, and the overall stability of the material (Figure 2f).

Designing the number of octahedral layers between organic spacers directly influences the electronic and optical properties of 2D perovskites since this thickness determines the confinement of charge carriers and quasi‐particles. For example, stacked structures with increasing layer thickness have been used to funnel excitons in the smallest bandgap region for enhancing emissive charge carrier recombination in LEDs (Figure 2g).^[^ ^53^, ^54^ ^]^ Finally, composition control, including interface passivation, doping, and ion exchange strategies, enables to modification of the cationic and anionic components of the MHPs structure, which can be used to create novel materials or to fine‐tune existing ones by reducing defects, or improving stability under operational condition (Figure 2h).

2

### 2.1 Heterostructures by Perovskite Phase Engineering

**Figure 3:**
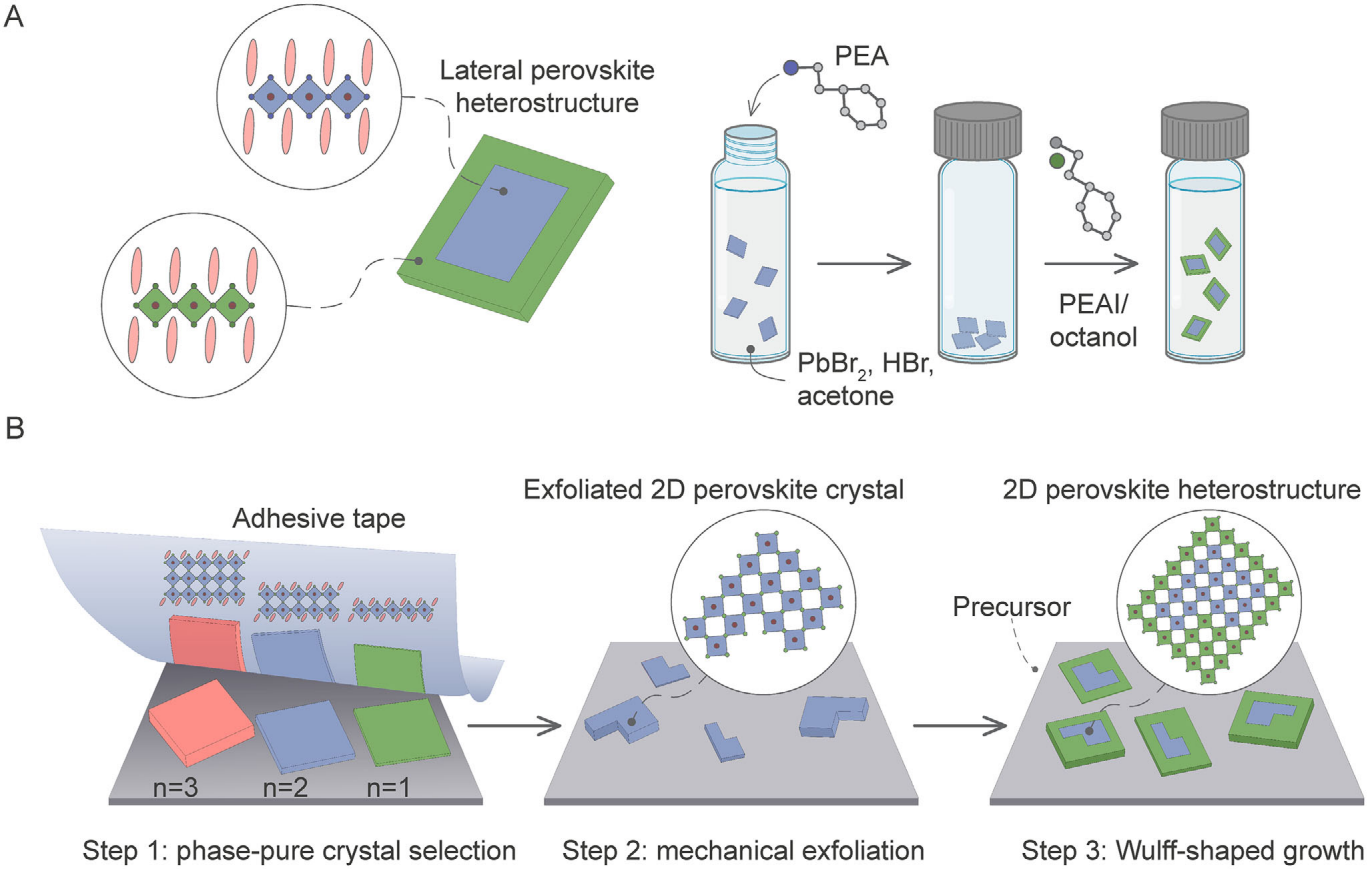
A) Microcrystalline heterostructure architecture and formation process of PEA_2_PbBr_4_‐PEA_2_PbI_4_ lateral heterostructures by a facile two‐step room temperature method. Adapted with permission from ref. [49] Copyright 2024, Wiley‐VCH. B) Schematic illustrations of kinetic Wulff‐shaped growth (3T)_2_PbI_4_–(3T)_2_SnI_4_ halide perovskite lateral heterostructure. Adapted with permission from ref. [66] Copyright 2025, Springer Nature.

MHPs have the great advantage that their optical properties (band gap, emission wavelength) can be readily tuned both by confinement effects and by their composition. In this section, we focus on heterostructures that originate from different compositions and discuss how they can affect charge carrier‐ and energy flow. Tuning the halide composition has proven to be a very successful approach to controlling the emission color.^[^ ^55^ ^]^ Heterojunctions with significant band offset can be fabricated by interfacing regions of different anion compositions, either by sequential growth or via post‐synthesis anion‐exchange processes. The latter strongly depends on anion diffusion in the materials, and in 3D perovskites or perovskite nanocrystals, anion diffusion leads mostly to alloyed phases.^[^ ^56^ ^]^ However, lower‐dimensional systems, such as 2D layered perovskites (2DLPs), exhibit tunable anion diffusivity due to the impermeability of the organic layer. This enables the formation of heterostructures composed of distinct halide phases, offering new possibilities for tailored optoelectronic properties.^[^ ^57^ ^]^ Vapor phase anion exchange was demonstrated in 2DLPs by exposing iodide microcrystals to hydrogen bromide vapors, which triggered anion exchange and resulted in the formation of a bromide‐phase shell.^[^ ^58^ ^]^ Our group extended this approach to solution‐based transformations in 2DLPs that can produce either microcrystals with a bromide phase core and iodide phase shell, or vice versa (**Figure** **3**a).^[^ ^49^ ^]^ Interestingly, we found that different exchange mechanisms are at work depending on the exchange route: dissolution‐recrystallization for bromide‐to‐iodide conversion and vacancy‐assisted diffusion processes for iodide‐to‐bromide exchange. The dependency of the exchange mechanism on the initial halide in lower‐dimensional perovskites can be attributed to the distinct positions that halide anions occupy within the octahedra.^[^ ^59^, ^60^ ^]^ While bromide preferentially occupies the equatorial position, iodide is predominantly located in the apical position. This selective occupation strongly impacts the formation of heterostructures in these systems via anion exchange. One plausible reason is that this preference is influenced by the organic layer and the resulting bonding environment, such as the number of hydrogen bonds formed or the type of perovskite‐forming headgroup positioned within the pocket.^[^ ^61^ ^]^ Additionally, the organic cation and its conformation in different halide phases may affect the solubility gap in the phase diagrams of these systems, thereby influencing the composition at the interface of the heterostructure. This effect will become less pronounced when the thickness of the inorganic layer increases and the architecture approaches the 3D structure.

2D materials with preferential in‐plane growth dynamics enable the formation of core‐crown geometries. The solution processability of perovskite materials has facilitated the fabrication of epitaxial lateral heterostructures through the controlled alternating growth of 2D perovskite phases using a sequential solvent‐antisolvent evaporation method. These multi‐step processes exploit the different solubility of the core and crown phases within a quaternary solvent system,^[^ ^25^ ^]^ which has enabled the formation of lateral heterostructures incorporating different halides (Br‐I) and metal cations (Pb‐Sn, Pb‐AgBi).^[^ ^25^, ^62^ ^]^ More recently, lateral heterostructures featuring distinct spacer cations have been realized with the same method.^[^ ^51^ ^]^ The choice of organic spacer cations influences octahedral distortion and can introduce additional functionality, such as the incorporation of organic semiconductors, further extending the tunability of these systems.^[^ ^63^, ^64^ ^]^ These structural variations highlight the versatility of organic‐inorganic design in tailoring perovskite properties at the molecular level. Moreover, spacer cation doping has been demonstrated as an effective strategy to stabilize polymorphs of 2DLPs that are otherwise only accessible under non‐ambient conditions, enabling the formation of homojunction with directional exciton diffusion.^[^ ^65^ ^]^ Although growing heterostructures between phases with *n* ≥ 2 is challenging due to solubility differences, recent advances using kinetic Wulff‐shaped growth allow precise control of slab thickness (n = 1–3) and modulation of the interfacial lattice mismatch while maintaining single‐crystal characteristics (Figure 3b).^[^ ^66^ ^]^ This was accomplished through the exfoliation of phase‐pure crystals, followed by controlled exposure to the growth solution, where careful tuning of the solvent system composition and growth time ensured selective phase formation.

A significant challenge in heterostructure growth is managing solubility differences, particularly when the core phase is more soluble in the chosen solvent system. This issue commonly arises in iodide‐ or tin‐based phases, which tend to dissolve more readily than their bromide‐ or lead‐based counterparts. Beyond the previously mentioned anion exchange approach for creating an iodide‐based core with a bromide‐based frame, careful selection of spacer cation combinations can further stabilize the core during growth. By using different spacer cations for the iodide core and bromide frame, solubility mismatches can be mitigated, allowing the Br frame to grow without dissolving or damaging the iodide core. Thus, identifying the optimal metal‐halide‐spacer combination is key to achieving controlled heterostructure formation within the selected solvent system.

Given the vast compositional space available for exploring potential heterostructures in lower‐dimensional MHP systems, a combination of efficient solution‐based anion exchange, and growth strategies combined with high‐throughput screening will accelerate the discovery of potential material combinations. Additionally, lab‐on‐a‐chip techniques, such as dip‐pen lithography, or microfluidics offer a precise and scalable approach for fabricating and investigating these heterostructures.^[^ ^67^, ^68^ ^]^ To further advance this field, in situ spectroscopy and imaging techniques can be integrated to elucidate growth mechanisms and identify key intermediate phases.^[^ ^49^, ^50^ ^]^ These efforts will contribute to establishing fundamental design principles for low‐dimensional heterostructures, bringing us closer to the rational design of next‐generation perovskite‐based materials.

The tunability outlined in this section enables precise control over band alignment, exciton diffusion, and charge carrier dynamics, which are crucial for optimizing light emission, energy transfer, and stability in optoelectronic devices. Together with solution‐based processing, these methods offer a scalable route for bandgap and charge transfer properties engineering, opening new avenues for optoelectronic applications in photodetectors and optical communication systems.

### 2.2 Design of Mixed‐Dimensional Metal Halide Perovskite Heterostructures

**Figure 4:**
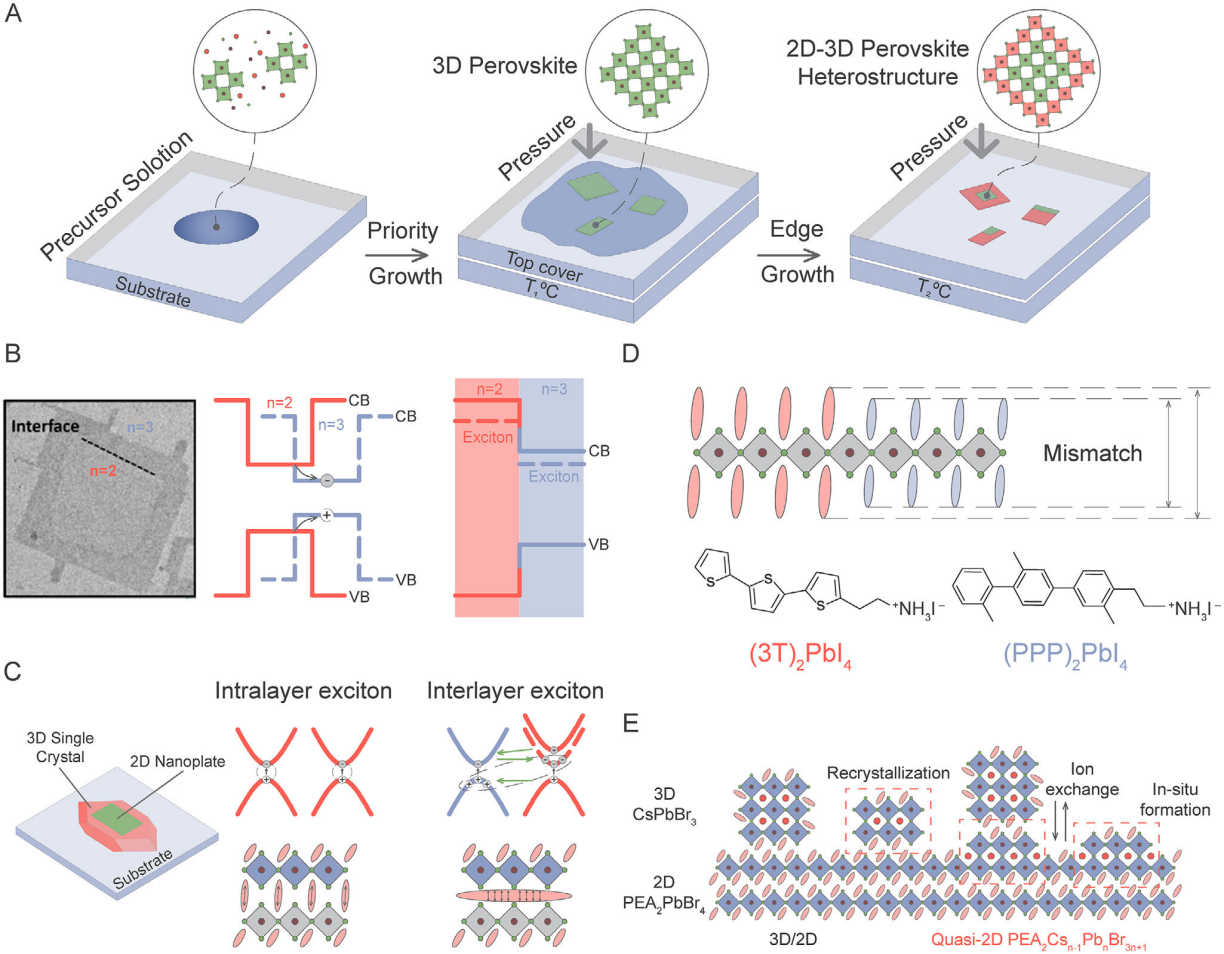
A) Schematic illustration of sequential growth of 2D‐3D perovskite lateral heterostructures by a one‐pot space‐confined method. Adapted with permission from ref. [69] Copyright 2025, Wiley‐VCH. B) Depiction of the coupled quantum‐well structure of the non‐integer CsPbBr_3_ 2D perovskite. Adapted with permission from ref. [71] Copyright 2023, American Chemical Society. C) Schematic model of the (BA)_2_PbBr_4_ nanoplate on 3D CsPbBr_3_ single crystal system and the scheme of formation of intra/inter‐layer exciton. Adapted with permission from ref. [70] Copyright 2025, Wiley‐VCH. D) Schematic illustration of a two‐dimensional perovskite lateral heterostructure. Adapted with permission from ref. [51] Copyright 2025, Springer. E) Scheme of oriented stacking of 3D NCs and quasi‐2D perovskite nanostructures on 2D NP. Adapted with permission from ref. [42] Copyright 2025, American Chemical Society.

Interfacing structures with different dimensionality provides exciting opportunities to design heterostructures that enable controlled energy flow. Such mixed‐dimensional heterostructures, for example, 2D and 3D hybrids, can potentially combine the advantages of 2D and 3D architectures in terms of carrier mobility, stability, and spectral bandwidth. For example, lateral integration of layered 2D perovskites with 3D perovskite crystals has yielded structures with dual‐band photoresponse and significantly enhanced detection performance.^[^ ^69^ ^]^ This approach leverages the stability and defect tolerance of 2D perovskites alongside the superior charge transport of their 3D counterparts. Remarkably, lateral growth techniques such as space‐confined methods enable the controlled synthesis of these heterostructures, facilitating their incorporation into wavelength‐tunable optoelectronic devices (**Figure** **4**a).

Energy funneling in MHP heterostructures has emerged as a powerful tool to manipulate excitonic behavior and enhance optoelectronic performance (Figure 4b). The development of non‐integer 2D semiconductor structures introduces a new paradigm in energy transfer mechanisms, as demonstrated in laterally coupled quantum well systems.^[^ ^17^ ^]^ These materials, exemplified by two‐dimensional cesium lead bromide perovskites with varying thickness regions, facilitate intra‐material energy migration without interfacial strain or dielectric barriers. This allows for unprecedented exciton funneling from thin to thick regions, as observed via time‐resolved spectroscopy, which can be leveraged for advanced light‐harvesting and emissive applications. Mixed‐dimensional heterostructures can interact with external fields and are intriguing because they host interlayer excitons. The distinctive optical properties, including prolonged exciton lifetime, enhanced energy transfer efficiency, and dual emission profiles, are attributed to the coexistence of intralayer excitons in the 2D layered perovskite and derived intralayer excitons from the (BA)_2_PbBr_4_ nanoplate on 3D CsPbBr_3_ single crystal system (Figure 4c).^[^ ^70^ ^]^ Additionally, lateral 2D perovskite heterostructure nanocrystals can accommodate distinct ligands across the junction despite significant lattice mismatch, forming ligand‐variant heterostructures. Interestingly, such heterostructures based on different organic cations can tolerate a large lattice mismatch of up to 16.5% in the out‐of‐plane direction, demonstrating remarkable structural flexibility. Their synthesis relies on a quaternary solvent system and sequential evaporation, ensuring controlled morphology and enabling tunable band alignment and charge carrier dynamics for optoelectronic applications (Figure 4d).^[^ ^51^ ^]^

Recent advancements in MHPs have demonstrated the potential of 2D/3D hybrid perovskite heterostructures for enhancing optoelectronic performance and environmental stability.^[^ ^72^ ^]^ However, challenges related to cation diffusion and phase transformation at 2D/3D interfaces remain a limiting factor. To address these issues, the real‐time structural dynamics of 3D/2D perovskite nano‐heterostructures, specifically CsPbBr_3_/PEA_2_PbBr_4_, were studied using in situ fluorescence spectroscopy. The results revealed dynamic cation migration and the formation of quasi‐2D phases at the interface, leading to optimized energy flow and the emergence of new electronic states. These findings provide new insights into the structural and optical evolution of 3D/2D perovskite heterostructures and suggest potential strategies for controlling energy transfer to improve optoelectronic device performance (Figure 4e).^[^ ^42^ ^]^

### 2.3 Carrier Transfer Dynamics in Mixed‐Dimensional Metal Halide Perovskite Heterostructures

Understanding and controlling carrier transfer dynamics in mixed‐dimensional MHP heterostructures is crucial for optimizing their optoelectronic performance. The interplay between dimensional confinement, interfacial coupling, and spacer composition governs the efficiency of charge and energy transfer processes.^[^ ^73^ ^]^ Structural parameters such as band alignment, layer thickness, and the nature of the heterointerface play a pivotal role in defining these processes, making precise interface engineering essential for modulating photophysical properties and designing device functionality. In this respect, recent studies have demonstrated that mixed‐dimensional perovskites offer a unique platform for tuning energy flow and exciton behavior through phase and dimensionality engineering.^[^ ^74^ ^]^

**Figure 5:**
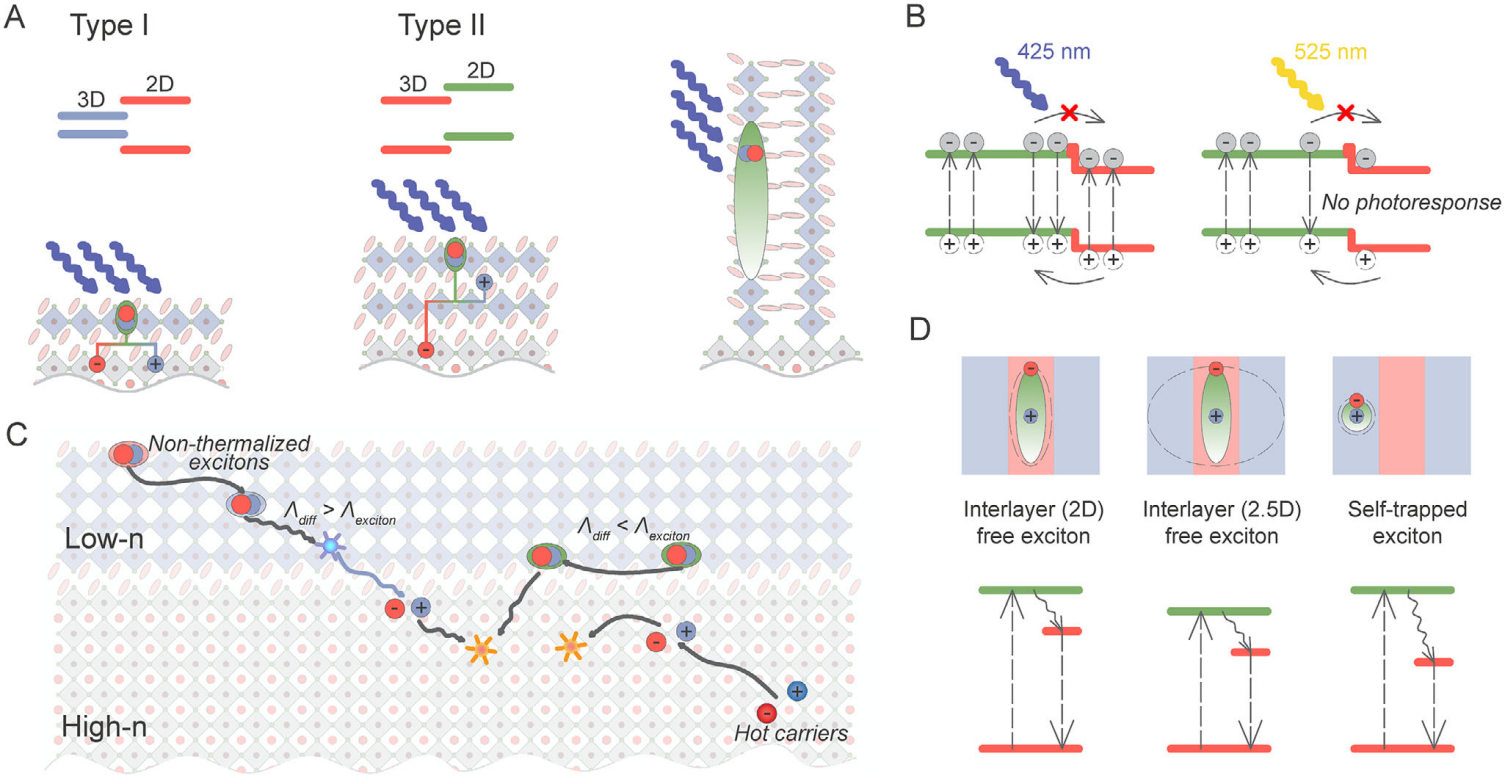
A) Horizontally and vertically aligned 2D perovskite plates on 3D perovskites, along with graphical representations of type‐I and type‐II kinetic models used to fit the time‐resolved PL dynamics of 2D/3D perovskite heterostructures. Adapted with permission from ref. [75] Copyright 2021, American Chemical Society. B) Energy band alignment diagrams of (BA)_2_MAPb_2_Br_7_‐MAPbBr_3_ under 425 and 525 nm illumination. Adapted with permission from ref. [81] Copyright 2024, Wiley‐VCH. C) Schematic representation of the photophysical processes occurring after excitation above the bandgap of all n‐phases in the film. Adapted with permission from ref. [82] Copyright 2024, Wiley‐VCH. D) Schematics of the energetic alignment of electronic states involved in PL1, PL2, and PL3 emission processes for monomethylhydrazinium lead bromide (MMHPbBr_3_) NC colloid, with insets illustrating the suggested excitonic nature of each emission band. Blue and gold represent the undistorted and distorted octahedral layers, while dashed and solid lines depict the presumed confinement potential wells, respectively. Adapted with permission from ref. [83] Copyright 2025, American Chemical Society.

Carrier transfer dynamics in 2D/3D perovskite heterostructures are strongly influenced by the band alignment type and the thickness of the 2D layer. In type‐I band alignment of bulk CsPbBr_3_ and (PEA)_2_PbBr_4_, the thin (PEA)_2_PbBr_4_ layers exhibit a delayed rise in the photoluminescence compared to CsPbBr_3_, indicative of efficient carrier transfer.^[^ ^75^ ^]^ As previously reported, energy transfer in such heterostructures occurs on a sub‐nanosecond timescale and can be modulated by tuning the A′‐site cations in 2D perovskite quantum wells, which control the spatial separation between donor and acceptor states and thus the efficiency of ultrafast FRET processes.^[^ ^76^, ^77^ ^]^ However, as the 2D layer thickness increases, a transition to type‐II alignment occurs, where electrons transfer to the 3D perovskite while holes remain localized in the 2D phase (**Figure** **5**a), leading to a reduced carrier transfer rate.^[^ ^78^ ^]^ This behavior highlights the influence of interfacial coupling and dimensional confinement on charge transport. Charge transfer is more efficient at oriented interfaces, where stronger interfacial coupling enhances energy flow control, resulting in longer carrier lifetimes compared to disordered interfaces. Such observations align with previous work highlighting the role of interfacial strain in modulating electronic structures and carrier dynamics, particularly in core/shell and layered perovskite systems.^[^ ^79^, ^80^ ^]^

In lateral (BA)_2_MAPb_2_Br_7_‐MAPbBr_3_ heterostructures, charge transfer behavior exhibits a strong dependence on the excitation wavelength, reflecting differences in band alignment (Figure 5b).^[^ ^81^ ^]^ Under 425 nm excitation, both 2D and 3D perovskite layers generate electron‐hole pairs, with holes efficiently crossing the interface while electrons face an energy barrier, thereby enhancing carrier collection and photocurrent. At 525 nm, where absorption primarily occurs in the 3D perovskite, the conduction band offset inhibits electron transfer, reducing overall efficiency. Beyond 600 nm, further reductions in interband absorption decrease the photoresponse. This spectral dependence illustrates how band engineering in lateral heterostructures can precisely control carrier dynamics, offering new opportunities for tunable optoelectronic devices. This behavior aligns with earlier findings that highlight the importance of dimensional heterogeneity in facilitating directional charge transport and improving device efficiency in multi‐phase perovskite systems.^[^ ^39^, ^84^ ^]^

An intriguing aspect of carrier dynamics in mixed‐dimensional MHPs is the relationship between structural flexibility and hot carrier cooling rates.^[^ ^85^ ^]^ While flexible crystal structures generally facilitate faster energy dissipation through lattice vibrations, the propylammonium (PA) film, despite its highly flexible structure due to a linear spacer and van der Waals gap, exhibits the slowest hot carrier cooling rate.^[^ ^82^ ^]^ In contrast, the phenylenedimethylammonium (PDMA) and PDMA‐PA films exhibit faster cooling rates. This counterintuitive behavior is in agreement with recent findings that Dion‐Jacobson MHPs exhibit stronger electron‐phonon interactions than RP MHPs due to additional phonon modes introduced by the organic spacer. Exciton transfer rates from low‐n (few octahedra layers sandwiched between organic → high confinement) to high‐n (many octahedra layers in between organic layers → low confinement) domains also vary with spacer composition, where rapid exciton transfer is critical to minimize energy losses in photovoltaic applications (Figure 5c). The PDMA film exhibits the fastest exciton transfer due to its conjugated structure, which promotes exciton delocalization, while the PA film shows the slowest rate, consistent with more localized exciton transport. Furthermore, a unique form of exciton confinement is observed in monomethylhydrazinium lead bromide (MMHPbBr_3_) NCs, where electrons are delocalized across the 3D lattice while holes remain confined within the 2D PbBr_6_ layer (Figure 5d).^[^ ^83^ ^]^ This 2.5D electronic structure differs from conventional 3D or 2D lead halide perovskites and provides new avenues for manipulating exciton behavior in optoelectronic devices. Colloidal MMHPbBr₃ NCs exhibit enhanced emission due to quantum confinement and effective surface passivation, unlike their bulk counterparts that are non‐emissive. These NCs exhibit size‐dependent absorption and PL, with two peaks corresponding to undistorted and distorted PbBr_6_ layers, indicative of electronic heterogeneity. Such 2.5D exciton confinement opens promising avenues for advanced applications in photonics, quantum devices, and tunable light‐emitting materials. This distinct exciton behavior aligns with recent observations of dual‐confinement effects in other hybrid perovskite nanostructures, where interface modulation enables tailored exciton properties for specific device applications.^[^ ^86^, ^87^ ^]^

The above‐discussed works collectively highlight the critical role of interface and spacer engineering in controlling carrier transfer dynamics in mixed‐dimensional perovskite heterostructures. By modulating band alignment, layer thickness, and structural flexibility, it is possible to fine‐tune charge and energy transfer processes to optimize performance across a range of optoelectronic applications. Continued exploration of these parameters will facilitate the development of next‐generation light‐harvesting and light‐emitting devices. A comprehensive understanding of the importance of structural and compositional tuning in enhancing the stability and efficiency of perovskite‐based materials will reinforce the value of dimensionality engineering in advanced optoelectronics.

### 2.4 Design of Heterostructures Beyond Perovskites: Exploring New Architectures for Energy Flow Control

**Figure 6:**
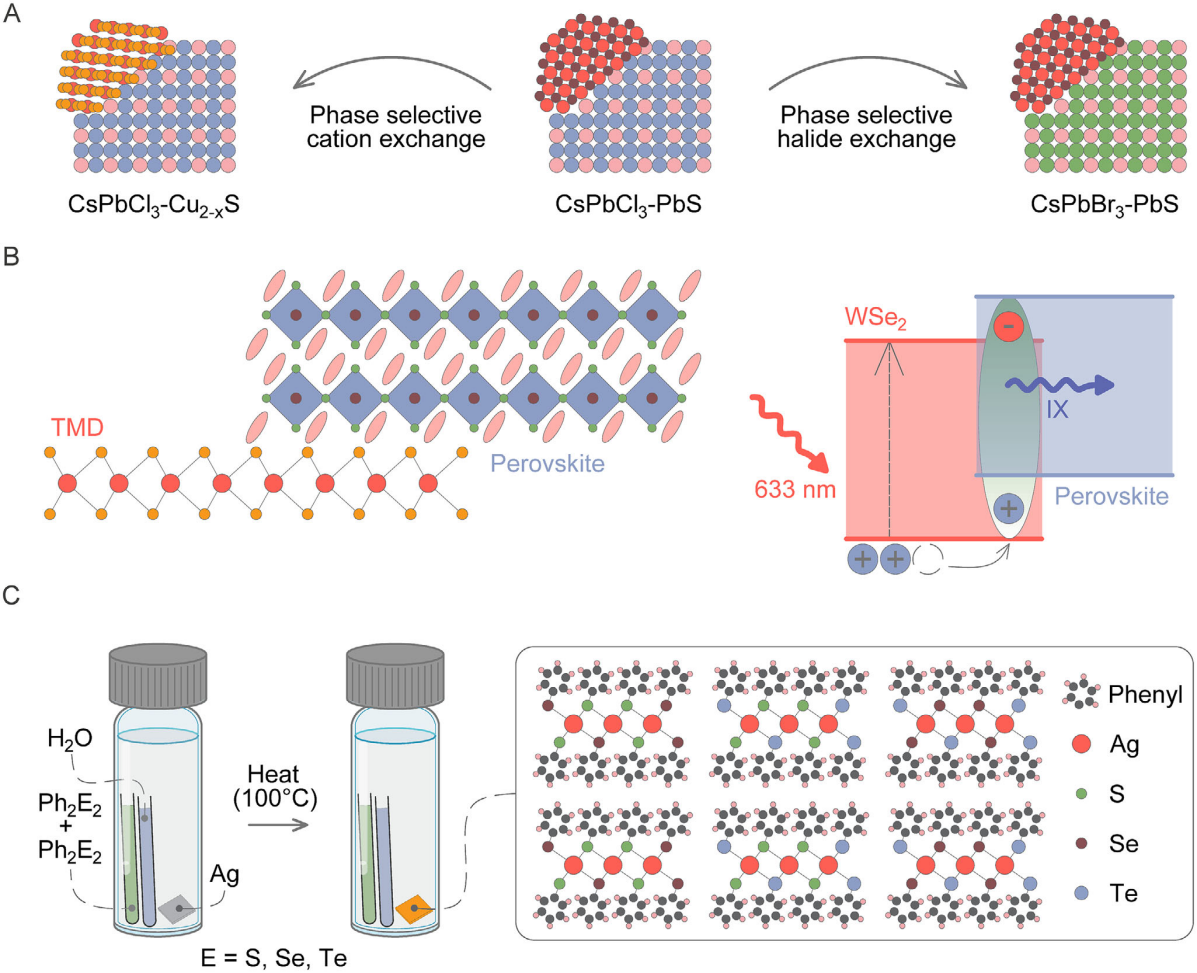
A) A scheme of Cl^–^ → Br^–^ and Pb^2+^ → Cu^+^ cation/anion exchange in nanocrystal heterostructures composed of CsPbCl_3_ and PbS domains sharing an epitaxial interface, yielding CsPbBr_3_–PbS and CsPbCl_3_–Cu_2‐_ *_x_* S epitaxial heterostructures, respectively. Adapted with permission from ref. [88] Copyright 2024, American Chemical Society. B) Schematic representation of a 2D perovskite/monolayer TMD heterostructure. The band alignment of the 2D perovskite/WSe₂ system forms a type‐II heterostructure with interlayer exciton (denoted as IX) emission under 633 nm laser excitation. Adapted with permission from ref. [92] Copyright 2020, American Chemical Society. C) A scheme of the tarnishing reaction to produce mixed‐chalcogen AgE_1‐x_E_x_Ph thin films (E=S, Se, Te). Adapted with permission from ref. [108] Copyright 2024, American Chemical Society.

Beyond single‐material systems, phase integration across different material classes offers a novel approach to multi‐material heterostructures. A recent example of this concept was demonstrated through epitaxial heterojunctions interfacing perovskite nanocrystals with metal sulfides. This was achieved by promoted PbS growth on CsPbCl₃ nanocrystals, followed by selective ion exchange reactions, enabling the controlled formation of CsPbBr₃‐PbS and CsPbCl₃‐Cu₂₋_2‐sx_S heterojunctions (**Figure** **6**a).^[^ ^88^ ^]^ These multidomain nanocrystals, composed of distinct material classes, introduce new possibilities for tailoring band alignment, emission properties, and charge carrier dynamics, further expanding the potential of hybrid heterostructures.

Similarly, lower‐dimensional perovskites, such as 2DLPs, offer a versatile platform for integration with other material classes, owing to their rich variety of tunable organic cations. Organic semiconductors and metal‐organic complexes exhibit similar molecular arrangements to the organic layers of 2DLPs, opening pathways for intergrowth or epitaxial growth on the 2DLP surface via solution‐ or vapor‐based methods. As recently demonstrated, the inorganic framework of 2DLPs can act as a structural backbone that guides the conformation of adjacent molecular layers, potentially enabling unique interactions with epitaxially grown organic semiconductors.^[^ ^89^ ^]^ Bifunctional organic molecules further expand this potential by enabling the formation of heterostructures without relying on lattice‐matching constraints. Instead, their integration is governed by compatible synthesis conditions that support the intergrowth of chemically distinct materials.^[^ ^90^, ^91^ ^]^ Vertically stacked van der Waals heterostructures combining 2DLPs with monolayer transition metal dichalcogenides (TMDs), such as MoS₂ and WSe₂, represent another promising strategy for multi‐material integration without the requirement of matching crystal structures (Figure 6b).^[^ ^92^ ^]^

These hybrid stacks can exhibit type‐II band alignment enabling the formation of interlayer excitons and offering new opportunities for low‐threshold lasing applications.^[^ ^93^ ^]^ While mechanically exfoliated flakes provide high‐quality materials, essential for fundamental studies, this method lacks scalability. However recent advances in high‐throughput mechanical exfoliation offer a promising route toward large‐area vertically stacked heterostructures.^[^ ^94^ ^]^

Research on perovskite‐based heterostructures has progressed in parallel with other systems, such as organic heterostructures (OHs) and other organic‐inorganic materials, which offer additional possibilities for tunable excitonic behavior and energy flow control. OHs, and particularly the perylene (Pe) and perylenecarboxaldehyde (PeO) systems, have gained significant attention due to their ability to precisely control exciton dynamics and photon transport.^[^ ^95^ ^]^ Heterostructures, synthesized via a two‐step approach combining liquid‐phase and vapor‐phase growth, show distinct optical behaviors that stem from the energy transfer between Pe and PeO molecules.

Another emerging class of materials with largely unexplored potential for heterostructures and mixed‐dimensional systems are metal‐organic chalcogenolates (MOCs). Like the more extensively studied 2DLPs, MOCs are layered 2D van der Waals semiconductors (Figure 6c). However, a key distinction between MOCs and 2DLPs is the presence of covalent bonding between the organic and inorganic layers, which significantly enhances their stability in both polar and nonpolar solvents. MOCs can be described by the general formula [M(ER)]_n_, where M═Cu(I), Ag(I), Au(I); E═S, Se, Te; and R is an organic moiety.^[^ ^96^ ^]^ Among the most studied MOCs is AgSePh, which exhibits blue photoluminescence (PL) and, due to its layered nature, possesses a high exciton binding energy, potentially leading to interesting light‐matter interactions. Recently, the field has gained momentum with new synthesis approaches, particularly for the growth of large single crystals, which are essential for structural investigations.^[^ ^97^ ^]^ Additionally, colloidal and quasi‐colloidal methods have been developed for synthesizing micro and nanometer‐sized crystals.^[^ ^98^, ^99^ ^]^ Beyond the above‐mentioned metals, Pb‐based organic chalcogenolates have also been synthesized, further expanding the material landscape. By changing the substitution group within the organic layer, it has been demonstrated that the local coordination environment of Pb can be adjusted from a hemidirected (asymmetric) to a holodirected (symmetric) coordination sphere, directly influencing the bandgap.^[^ ^100^ ^]^ Furthermore, introducing electron‐donating groups into the organic layer can lead to bandgap narrowing, while the incorporation of heterocyclic molecules enables dimensional engineering, both offering exciting opportunities for fine‐tuning the optical properties of these materials.^[^ ^101^, ^102^ ^]^ Recently, alloying in the AgSePh–AgTePh system revealed a fascinating competition between delocalized and self‐trapped excitons, paving the way for precise control of energy flow in these materials.^[^ ^103^ ^]^

Mixed‐dimensional architectures in such emerging materials can offer novel solutions for controlling energy flow and excitonic behavior. These systems provide an exciting opportunity to design highly tunable optoelectronic materials, opening avenues for applications in flexible electronics, quantum information processing, and energy‐efficient photonic technologies and energy‐efficient photonic technologies.^[^ ^104^, ^105^, ^106^ ^]^ The molecular design of the organic layer is central to realizing these architectures, as it governs interfacial properties and enables the formation of complex heterostructures of mixed‐dimensional systems. While straightforward synthesis strategies, similar to those used in perovskite‐based systems, are still in their infancy, they are essential for exploring the vast compositional space and identifying promising material combinations. Although heterostructures in MOCs have yet to be realized, their feasibility is supported by the success of other low‐dimensional organic‐inorganic materials, such as 2DLPs. In this context, the AgSePh‐AgTePh system presents an intriguing starting point for developing, e.g., lateral heterostructures, given the compatibility of their crystal structures.^[^ ^107^ ^]^

## 3 Future Outlook

**Figure 7:**
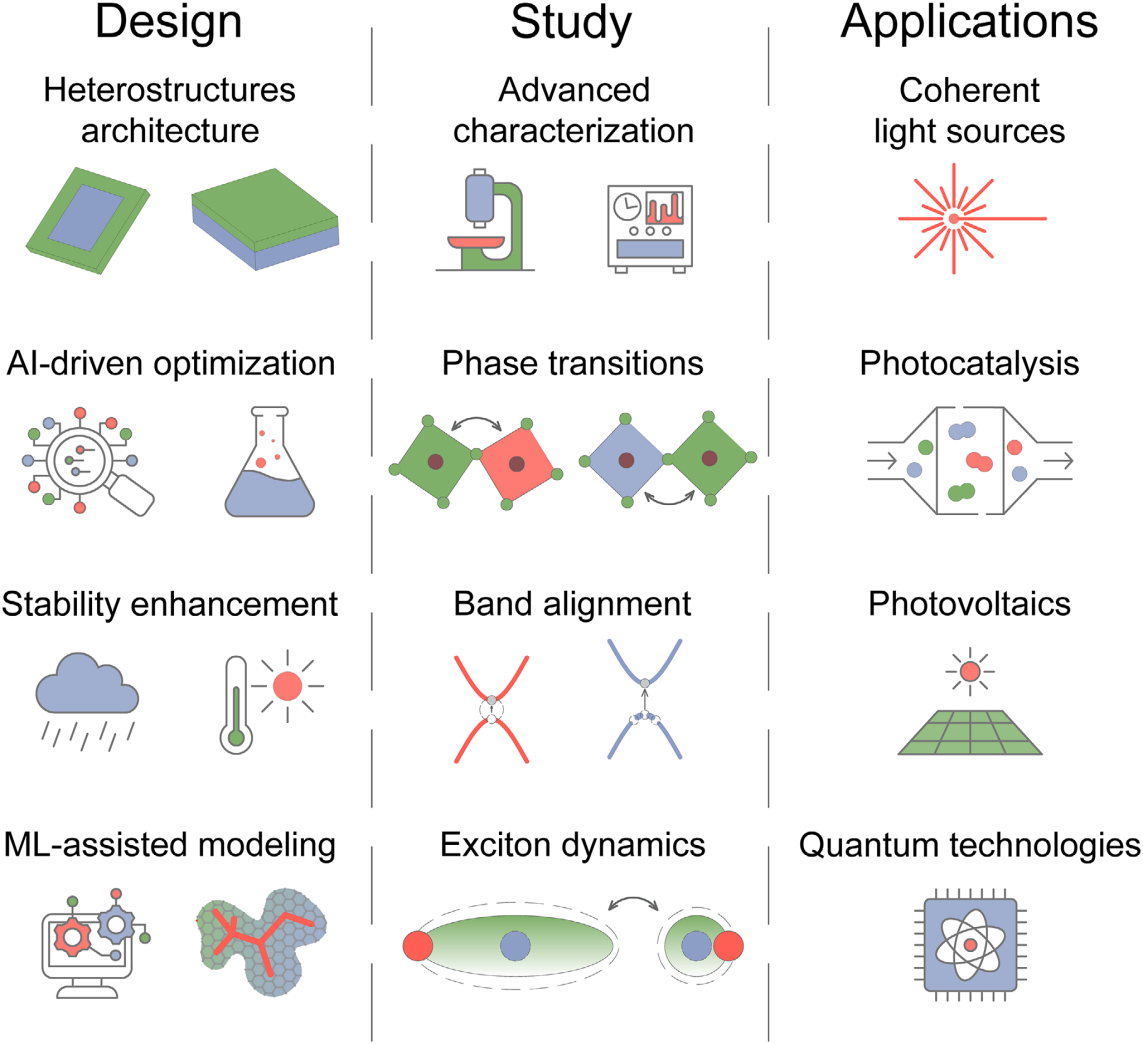
Roadmap that illustrates how innovative architectures and tools (Design) combined with a deep understanding of the fundamental mechanisms (Study) governing energy transfer in perovskite heterostructures can unlock their potential in next‐generation optoelectronic and quantum devices (Applications).

The control of energy flow in perovskite heterostructures through phase and dimensionality engineering represents a rapidly evolving frontier in materials science with profound implications for next‐generation optoelectronic applications. **Figure** **7** illustrates how the strategic design of heterostructure architectures, coupled with data‐driven optimization and targeted stability enhancement, forms the basis for rational material engineering. These design efforts must be supported by advanced characterization techniques and machine learning‐assisted modeling to uncover the local structural dynamics and interfacial phenomena. Investigating phase transitions, band alignment, and exciton behavior enables precise control over energy flow at the nanoscale. By mastering these mechanisms, perovskite heterostructures can be tailored for a broad spectrum of cutting‐edge applications, including coherent light sources, photocatalysis, photovoltaics, and quantum technologies, extending far beyond traditional solar cells and light‐emitting diodes. Another highly interesting frontier will be the ability to induce and control dynamic phase transitions in these materials that offer unique opportunities for developing switchable and tunable optoelectronic devices.

To move toward these goals from the experimental side, leveraging on the interplay between different perovskite phases and dimensional regimes allows to tailor charge transport, exciton dynamics, and radiative recombination processes, which enables the design of materials with enhanced emission or charge separation efficiency, stability, and functionality. The integration of mixed‐dimensional MHP structures allows for the precise management of exciton confinement and energy or charge transfer pathways. This multidimensional approach has led to significant advancements, including improved light emission through energy‐cascading mechanisms and enhanced carrier extraction via interfacial layers. Considering that exciton diffusion length and mean free carrier paths in these materials are in the submicron range, future research should aim at compatible crystal dimensions to optimally exploit these features, for example in photocatalysis, light emission management, sensing and energy harvesting. Similarly, there will be a growing need to investigate such processes with high spatial resolution, for example, to map anisotropic exciton diffusion across such heterointerfaces. Since structure strongly defines function, there will be a need for combined structural‐optoelectronic investigation methods, and here the combination of optical spectroscopy with electron microscopy such as time‐resolved photoemission electron microscopy (TR‐PEEM), ultrafast electron diffraction (UED), and cathodoluminescence should provide very promising avenues.

Key areas for future research include the role of defect states, interfacial strain, and exciton binding energies in understanding charge carrier dynamics. In this respect, managing the possibly large lattice mismatch across heterojunctions is essential for ensuring stable heterostructure formation. Here, the use of specific organic ligands not only facilitates type‐I but also enables type‐II energy alignments that can be employed toward precise control over optical properties and allows for adaptive energy band alignment.

Interface properties play a critical role in charge carrier dynamics, requiring multifunctional tools that combine high penetration depth for interface access with the potential for atomic‐level structural resolution, while also enabling to probe of optical and electrical properties. Here, in addition to the techniques mentioned above, advanced spectroscopic methods such as transient absorption, time‐resolved photoluminescence, impedance spectroscopy, and deep‐level transient spectroscopy will be highly relevant. Scanning probe techniques like Kelvin probe force microscopy (KPFM) and conductive atomic force microscopy (C‐AFM) can also provide access to the Fermi level, work function, and thus insights into band alignment and charge transport at the interface. Future studies of perovskite heterojunction interfaces will focus on correlating structural, optical, and electronic properties with nanoscale precision, enabling a deeper understanding of how interfacial strain, defect states, and band alignment govern charge transport and recombination dynamics. Advancing methods that combine high spatial, temporal, and spectral resolution will be essential for unveiling the complex interplay between structure and function in these materials.

From a theoretical and modeling point of view, the mixed‐phase or mixed‐dimensionality structures that we considered have mostly mesoscopic length scales, and therefore precise modeling by first‐principles methods is either impossible or computationally expensive. Therefore, approaches that combine density functional theory with artificial intelligence (AI) or machine learning models can present viable methods to describe such materials in sufficient detail to make precise predictions on their properties and behavior. Recent AI‐driven studies have demonstrated the power of data‐guided screening strategies, such as selecting electronically active organic spacers in layered Dion–Jacobson perovskites to induce type‐II heterojunctions with efficient interfacial charge transfer.^[^ ^109^ ^]^ In parallel, the integration of high‐throughput first‐principles calculations with crystal graph neural networks has enabled the rapid prediction of band alignments in thousands of MHPs heterostructures, revealing promising candidates for solar cells and photocatalysis.^[^ ^110^ ^]^ Furthermore, AI‐driven research frameworks, such as predictive modeling tools, can streamline the research process by reducing reliance on traditional trial‐and‐error experimentation. By harnessing machine learning algorithms to analyze experimental data and identify trends, researchers can accelerate the discovery of novel materials and device architectures.

## Conflict of Interest

The authors declare no conflict of interest.
